# Difficulties in the differential diagnosis of large solitary pulmonary cysts

**DOI:** 10.1093/icvts/ivab292

**Published:** 2021-10-30

**Authors:** Anna Elisabeth Frick, Hendrik Jan Ankersmit, Ingrid Simonitsch-Klupp, Konrad Hoetzenecker

**Affiliations:** 1 Division of Thoracic Surgery, Department of Surgery, Medical University of Vienna, Vienna, Austria; 2 Department of Pathology, Medical University of Vienna, Vienna, Austria

**Keywords:** Pulmonary cyst, Squamous cell carcinoma, Lobectomy, Pulmonary cavity

## Abstract

Large solitary cystic lesions are a rare finding, and their differential diagnosis includes cystic airspaces associated with lung cancer, congenital pulmonary airway malformations and pneumatoceles. Here, we report 3 consecutive patients who presented with a large solitary pulmonary cyst on chest computed tomography. All underwent surgical resection, and the histopathological findings were different in all 3 cases. In one patient, a very rare finding of squamous cell carcinoma arising from the cystic lesion in the left lower lobe was confirmed. Therefore, in carefully selected cases, pulmonary cysts should be resected based on the potential risk for recurrent infection and the development of malignancy.

## INTRODUCTION

Pulmonary cysts are thin-walled (<4 mm) gas or fluid-filled structures within the lung parenchyma. Differential diagnosis is based on the cyst’s size, inner wall contour, contents, distribution and clinical presentation. Most patients with pulmonary cysts are asymptomatic, but common clinical symptoms are cough, recurrent pneumonia, dyspnoea and spontaneous pneumothorax [1]. A retrospective analysis of 34 801 computed tomography (CT) scans in 2954 patients with non-small cell carcinoma found that 1% of cases were associated with cystic airspaces, which were more frequently associated with adenocarcinomas than squamous cell carcinoma [2]. Solitary pulmonary cavities with a wall thickness >15 mm are malignant in 95% of cases, and thin-walled cysts are benign in 92% of cases [3]. However, the probability of incidental findings of asymptomatic pulmonary cysts is increased with the widespread use of CT.

Here, we present 3 consecutive patients diagnosed with a large solitary pulmonary cyst and discuss the difficulties in reaching a differential diagnosis based on their histopathological findings.

## CASE PRESENTATIONS

### Case 1

**Figure 1:**
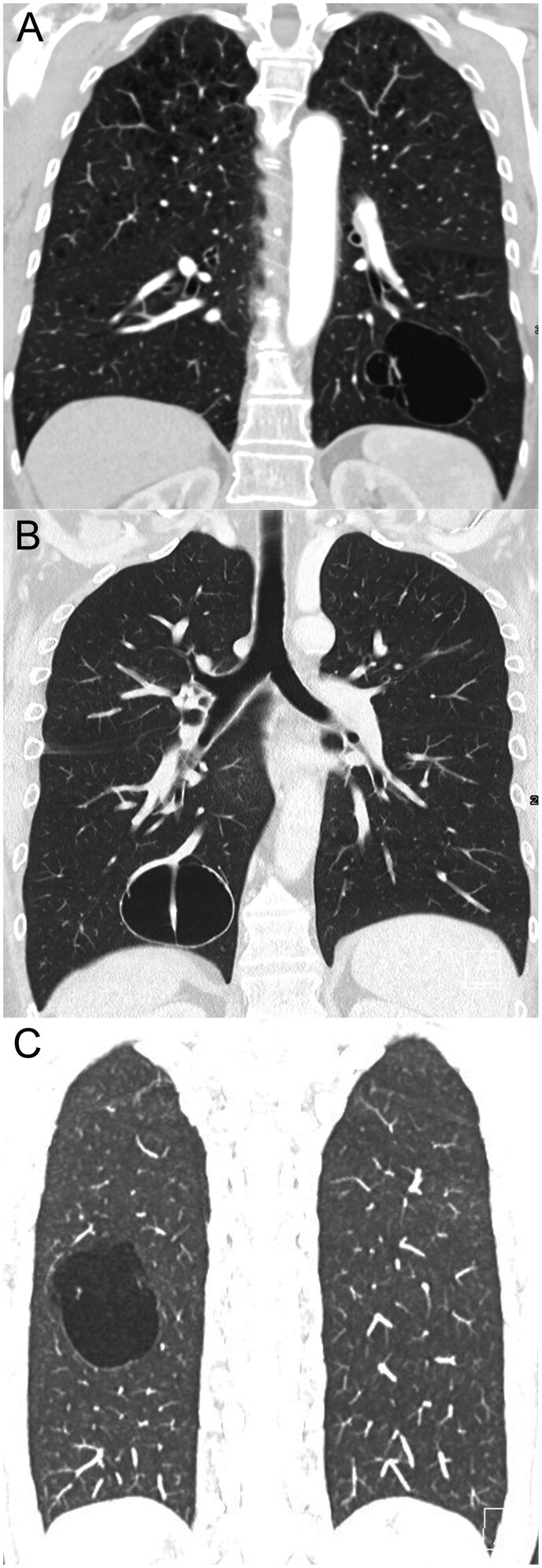
Chest computed tomography (CT) in coronal view. (**A**) In Case 1, contrast-enhanced CT showed a thin-walled lobulated cyst in the left lower lobe with some thin septae. There are no solid components and no mediastinal or hilar lymphadenopathy. (**B**) In Case 2, CT demonstrated a right lower lobe cyst with a thick wall (>4 mm). (**C**) In Case 3, CT showed a thin-walled (<4 mm) cyst in the right lower lobe.

A 47-year-old female presented to our outpatient clinic with a large pulmonary cyst in the left lower lobe but no symptoms. The patient’s medical history was unremarkable except a moderate smoking history (20 pack-years). Contrast-enhanced chest CT showed a 5-cm diameter, thin-walled air-filled cyst in the left lower lobe (Fig. 1A).

**Figure 2:**
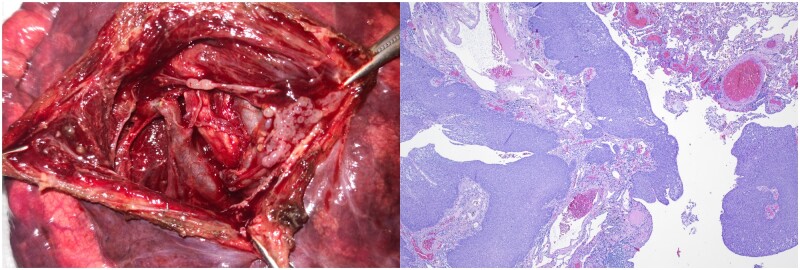
Macroscopic and microscopic images of a suspicious cyst in Case 1. (**A**) Intraoperatively opened cyst with suspicious nodular changes. (**B**) Microscopic examination revealed squamous cell carcinoma in situ with focal minimal ongoing stromal invasion.

After interdisciplinary discussion between the thoracic surgeon, pulmonologists and radiologists, surgical resection of the cyst was offered to the patient, who insisted on a surgical procedure. Left posterior muscle-sparing thoracotomy was performed in the auscultatory triangle (fifth intercostal space; 6 cm) without dividing any of the major chest wall muscles, and appropriate mobilization of the muscles allowed full access. Intraoperatively, the cyst was opened, revealing suspicious atypical nodular changes. A frozen section revealed squamous cell carcinoma (Fig. 2A). Therefore, lobectomy of the left lower lobe with additional radical lymphadenectomy was performed.

Macroscopic inspection of the resected lobe revealed a 4-cm diameter cyst at the base of the lobe. The periphery of this area contained polycyclic ridge-like tissue masses. Histology revealed extensive bronchiectatic lung tissue; the extended sack-shaped bronchial structures were lined by atypical squamous epithelium with focal stromal invasion (Fig. 2B). Thus, squamous cell carcinoma *in situ* with focal minimal ongoing stromal invasion pT1a, arising in a wide-ranging bronchiectasia, was diagnosed.

The postoperative course was uneventful, and the patient has been regularly followed up for 391 days without evidence of cancer relapse.

### Case 2

A 49-year-old male was diagnosed with a slowly growing cyst in the right lower lobe. The cyst was followed with regular CT scans over 6 months (Fig. 1B). The patient had a known drug addiction and cardiac comorbidities, including aortic regurgitation grade II, hepatitis C and a suspicious liver lesion. The tumour board suggested positron emission tomography–computed tomography, but no hypermetabolic tracer uptake was observed in the liver or lung lesions. Because adenocarcinoma of the lung could not be ruled out and the cyst was 6 cm in diameter, the right lower lobe cyst was resected.

The patient underwent a right-sided video-assisted thoracoscopic surgery procedure but, due to difficulties with multiple adhesions and the risk of postoperative bleeding and complications from his comorbidities, fast conversion to anterior muscle-sparing thoracotomy was considered. Wedge resection of the cystic formation of the right lower lobe was performed. The frozen section revealed a benign cystic process, and no further surgical resection was needed.

Histology revealed non-necrotizing granulomatous interstitial pneumonia with foreign body reaction and bronchitis. The postoperative course was unremarkable; the patient was discharged on the fourth postoperative day, requiring no further treatment.

### Case 3

A 25-year-old female presented with recurrent right-sided pneumothorax previously treated twice with a chest tube, fully expanding the lung. Her remaining medical history was unremarkable.

Chest CT showed a 5-cm diameter, thin-walled pulmonary cyst in the right lower lobe (Fig. 1C). Due to the recurrent pneumothorax, right-sided video-assisted thoracoscopy with wedge resection of the apical right upper lobe, radical parietal pleurectomy and resection of the right lower lobe cyst was performed. A frozen section of the right lower lobe cyst revealed a benign pulmonary cystic process. Histology showed a 1.8-cm thin-walled cystic lesion with a smooth inner surface wall and cystic structures lined by bronchiolar ciliated epithelium, without any atypia. The cyst wall comprised smooth muscle and elastic fibres. The diagnosis of congenital pulmonary airway malformation (CPAM) type 2 was established.

The postoperative course was uneventful and the patient was discharged on the fifth postoperative day with a fully expanded lung. No further treatment was required.

## DISCUSSION

We described 3 consecutive cases that exemplify the difficulties in achieving a differential diagnosis for large solitary pulmonary cysts.

Case 1 represented a very rare finding of squamous cell carcinoma arising from an extensive thin-walled pulmonary cyst in an asymptomatic patient. The differential diagnosis in this particular case included CPAM 1, pneumatocele, giant bulla (i.e. sharply demarcated areas of emphysema with a wall thickness <1 mm), pulmonary sequestration and congenital lobar emphysema. Due to the size of the cyst, absence of lung infection and presence of healthy surrounding parenchyma, pneumatocele was excluded. Considering the potential malignant transformation of CPAM 1 and risk of infection, surgical resection was a therapeutic option.

Case 2 was a patient with non-necrotizing granulomatous interstitial pneumonia. Granulomatous lung diseases are common pulmonary abnormalities with variable clinical manifestations; diagnosis is based on clinical and radiological findings. Typical causes of necrotizing granulomas are fungal or parasitic infections. However, non-necrotizing granulomas of sarcoidosis are described as discrete, well-formed and interstitial. In cases of aspiration or intravenous drug use, peribronchiolar or perivascular distribution of foreign material and non-necrotizing granuloma should be considered [4]. Considering the possible differential diagnosis and potential risk for malignancy, surgical resection of the cystic formation was indicated.

Case 3 was a young patient with recurrent right-sided pneumothorax and a pulmonary thin-walled cyst in the right lower lobe on CT. The cyst was revealed to be CPAM 2, a rare cystic anomaly occurring during foetal development with a risk of recurrent infections. Surgical resection of asymptomatic cysts remains controversial, but the decision for surgical resection was based on the recurrent pneumothorax.

In the final pathology of all 3 cases, the size of the lesion always measured smaller than on the CT scan, probably due to the known shrinkage effect of formalin fixation and histological processing. Pulmonary cysts occur frequently and the incidence is increasing in asymptomatic individuals >40 years old and could be part of the ageing process [5].

In conclusion, we described 3 different clinical scenarios and surgical interventions for asymptomatic pulmonary cysts with difficult differential diagnoses. The remaining question for pulmonary cysts is whether surgical resection is indicated in asymptomatic patients. Careful selection considering the risks and benefits is crucial.

**Conflict of interest:** none declared. 

## Reviewer information

Interactive CardioVascular and Thoracic Surgery thanks Paola Ciriaco and the other anonymous reviewers for their contribution to the peer review process of this article.
